# Exact replication: Foundation of science or game of chance?

**DOI:** 10.1371/journal.pbio.3000188

**Published:** 2019-04-09

**Authors:** Sophie K. Piper, Ulrike Grittner, Andre Rex, Nico Riedel, Felix Fischer, Robert Nadon, Bob Siegerink, Ulrich Dirnagl

**Affiliations:** 1 Institute of Biometry and Clinical Epidemiology, Charité—Universitätsmedizin Berlin, corporate member of Freie Universität Berlin, Humboldt-Universität zu Berlin, and Berlin Institute of Health, Berlin, Germany; 2 Berlin Institute of Health (BIH), Berlin, Germany; 3 Center for Stroke Research, Charité—Universitätsmedizin Berlin, corporate member of Freie Universität Berlin, Humboldt-Universität zu Berlin, and Berlin Institute of Health, Berlin, Germany; 4 Department of Experimental Neurology, Charité—Universitätsmedizin Berlin, corporate member of Freie Universität Berlin, Humboldt-Universität zu Berlin, and Berlin Institute of Health, Berlin, Germany; 5 Berlin Institute of Health—QUEST The Center for Transforming Biomedical Research, Charité—Universitätsmedizin Berlin, corporate member of Freie Universität Berlin, Humboldt Universität zu Berlin, and Berlin Institute of Health, Berlin, Germany; 6 Department of Psychosomatic Medicine, Center for Internal Medicine and Dermatology, Charité—Universitätsmedizin Berlin, corporate member of Freie Universität Berlin, Humboldt-Universität zu Berlin, and Berlin Institute of Health, Berlin, Germany; 7 McGill University and Genome Quebec Innovation Centre, McGill University, Montreal, Canada; 8 Department of Human Genetics, McGill University, Montreal, Canada; 9 German Center for Neurodegenerative Diseases (DZNE), Berlin Site, Berlin, Germany; 10 German Center for Cardiovascular Research (DZHK), Berlin site, Berlin, Germany; 11 NeuroCure Clinical Research Center, Charité—Universitätsmedizin Berlin, corporate member of Freie Universität Berlin, Humboldt-Universität zu Berlin, and Berlin Institute of Health, Berlin, Germany; University of California San Francisco, UNITED STATES

## Abstract

The need for replication of initial results has been rediscovered only recently in many fields of research. In preclinical biomedical research, it is common practice to conduct exact replications with the same sample sizes as those used in the initial experiments. Such replication attempts, however, have lower probability of replication than is generally appreciated. Indeed, in the common scenario of an effect just reaching statistical significance, the statistical power of the replication experiment assuming the same effect size is approximately 50%—in essence, a coin toss. Accordingly, we use the provocative analogy of “replicating” a neuroprotective drug animal study with a coin flip to highlight the need for larger sample sizes in replication experiments. Additionally, we provide detailed background for the probability of obtaining a significant *p* value in a replication experiment and discuss the variability of *p* values as well as pitfalls of simple binary significance testing in both initial preclinical experiments and replication studies with small sample sizes. We conclude that power analysis for determining the sample size for a replication study is obligatory within the currently dominant hypothesis testing framework. Moreover, publications should include effect size point estimates and corresponding measures of precision, e.g., confidence intervals, to allow readers to assess the magnitude and direction of reported effects and to potentially combine the results of initial and replication study later through Bayesian or meta-analytic approaches.

## Abstract 2

Using a coin toss to ‘replicate’ a neuroprotective effect of valproic acid, this study highlights the fact that exact replications of biomedical experiments are usually underpowered, often with power of approximately 50%.

## Introduction

“Non-reproducible single occurrences are of no significance to science.” [1].

In modern times, replication of results has been considered an integral part of the scientific process, at least, since Karl Popper’s famous declaration [2], and has again taken center stage in discussions about current research and publication practices. Among the life sciences, psychology was the first field to attempt large scale replications of key research findings [3–5], with discouraging results. Successful replications in these three multiexperiment metastudies varied from 39% to 67%, depending on the study and how replication was defined. An initially similarly large scale (but now much reduced) replication study in cancer biology [6] has produced mixed results and has encountered difficulties in conducting replications, in part due to lack of methodological details in the original papers and the unavailability of reagents from the original labs. Anecdotal evidence from the pharmaceutical industry, however, suggests that exact replication success in the related field of drug development is low, found to be 11% by Begley and Ellis [7] and 26% by Prinz and colleagues [8].

As a consequence, many biomedical researchers are aware of potentially low replication rates across laboratories [9]. Largely unappreciated, however, are the potentially low replication rates of exact replications (also called “strict replications”) within laboratories, in which experiments are repeated with new samples with the same protocols and sample sizes. Unbeknownst to most researchers, however, using sample sizes identical to those of the initial experiments usually results in statistically underpowered replication attempts. At the extreme, the probability of obtaining a significant result in an exact replication of an initially barely significant result can be close to that of a coin toss [10].

We use an empirical example from our own research to highlight the generally low statistical power of same sample-size exact replications, with emphasis on the common scenario of a barely significant initial finding. Unconventionally to this end, we conduct a coin flip experiment in an attempt to “replicate” an animal experiment that found a small neuroprotective effect of valproic acid (VPA). We use this admittedly absurd procedure to provide the background for a broader discussion of the caveats and challenges implicated in replications of preclinical experiments. In particular, we discuss the variability of *p* values in replication attempts as well as the pitfalls of simple binary (significant or not significant) testing.

## The initial experiment

VPA has been widely used as an anticonvulsant and mood-stabilizing drug for the treatment of epilepsy and bipolar disorders. Additional uses of the drug have been suggested by studies that have demonstrated its neuroprotective properties in rats [11, 12]. Results from our group suggested such a protective effect of VPA in reducing brain infarct volumes in mice.

In the experiment, 20 male C57Bl/6 N mice underwent transient intraluminal middle cerebral artery occlusion (MCAO) for 45 minutes (for detailed description, see S1 Text). Ten mice administered VPA (30 mg/kg, i.p., Desitin, Hamburg, Germany) were compared to 10 animals administered vehicle only. Delivery was done immediately after reperfusion, 12 hours later, and then twice daily (every 12 hours) for 7 days. The primary outcome of interest was brain infarct measured in mm^3^. The VPA treated group displayed significantly lower infarct volumes (−37%) compared with the vehicle treated group (mean: 39.4 mm^3^, standard deviation [SD]: 27.6 mm^3^ versus 63.6 mm^3^, SD: 22.7; *n* = 10 per group; mean difference: 24.2 mm^3^ with 95% confidence interval [CI; 0.3–48.0 mm^3^]; standardized effect size of 0.96 (95% CI: 0.01–1.87); t = 2.136; *p* = 0.047; see S1 Fig).

### Ethics statement

All animal experiments, inclusive of the welfare-related assessments and interventions that were carried out prior to, during, or after the experiment, were performed according to protocols approved by the Berlin Authorities (ethics committee of the “Landesamt für Gesundheit und Soziales Berlin,” LaGeSo Reg 390/09).

## Replacing mice with dice?

Given that the *p* value from the initial experiment was only slightly less than 0.05, we had initially planned to replicate our finding with a new mouse experiment. Assuming that the observed group means and their pooled SD equal the true population values, a formal power calculation for an exact replication (t-test for independent groups with a 0.05 two-sided alpha level and the same sample size of *n* = 10 per group) yields a power of 52% (nQuery Advisor 7.0, Statistical Solutions Ltd, Cork, Ireland), i.e., approximately that of a coin flip.

Rather than conduct a same sample-size exact replication as is typically done in biomedicine, however, we decided to attempt to “replicate” our initial findings by a probabilistically equivalent Bernoulli experiment—a simple coin flip (see Box 1 for a more detailed rationale). It should be noted that, as is true for any power estimation, our assumption that the observed effect in the initial experiment equals the population effect cannot be tested (i.e., the true population effect may be smaller or larger). Notwithstanding, this assumption is routinely used for power calculations in applied contexts because it is typically the best estimate available in preclinical research.

**Box 1. Probability of successful replication**

For a repetition of the experiment with the same sample size, intervention, and groups, the probability of again obtaining a significant result is equal to the power of the replication experiment with respect to identifying the observed effect size from the first experiment [10]. In the case of a t-test, the distribution of future results will follow a noncentral Student t-distribution with N_total_-2 degrees of freedom and a noncentrality parameter (ncp) that depends on the observed effect d of the original study and the size n of each group: ncp = d*√(n/2). For a single duplicate experiment, the probability of a statistically significant result in in the same direction as the original experiment corresponds to the area Φt under the curve of the density function of the t distribution beyond the critical value tα/2 for the corresponding alpha error probability. In our original study, we had an effect size d of 0.957 with *n* = 10 per group corresponding to a ncp of 2.140 and a critical value tα/2 (for α = 0.05) of 2.101, resulting in a probability of successful replication at the same alpha level of Φt (2.101, 2.140, df = 18) = 0.526. Because it is specified as a replication experiment, this result does not depend on the power of the original study [10].

**Fig 1:**
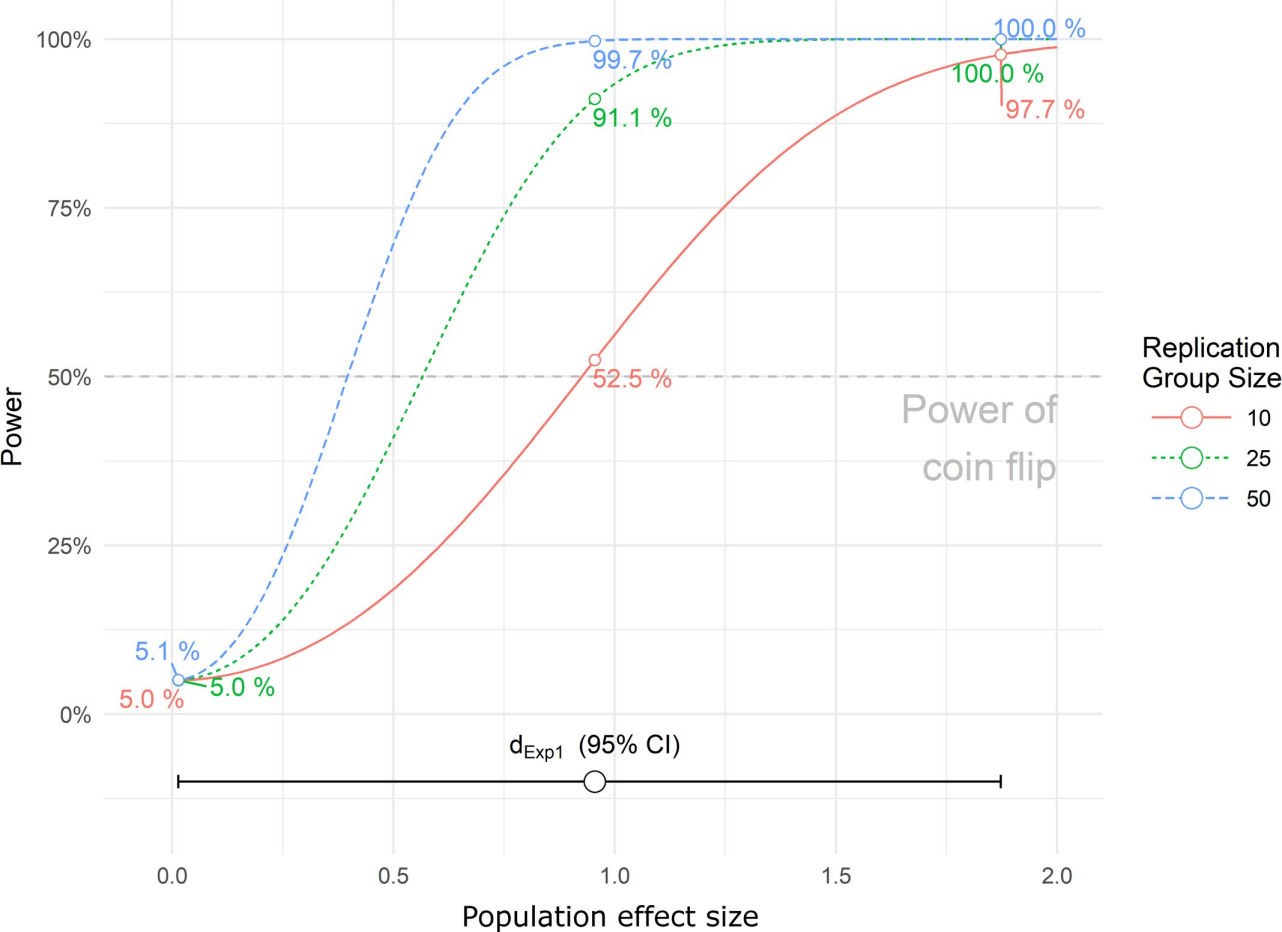
Power of replication experiment depending on the expected effect size and sample size. Colored numerical values refer to our original experiment. Data of this figure can be found in S1 Data, and the figure can be explored further under s-quest.bihealth.org/power_replication/.

A caveat is needed regarding the coin flip analogy used in our study. The analogy only holds under the assumption that the estimated effect size in the first experiment equals the true population effect. In general, however, data from initial experiments are consistent with a broad range of effect sizes, as can be inferred from the wide confidence intervals associated with the effects. Fig 1 shows the power of a replication experiments with three different sample sizes as a function of expected effect. Note that in our example, which has an initial *p* less than but close to 0.05), tossing a coin has approximately the same power as an exact replication to detect an effect size as large as in experiment 1. The true population effect is unknown, however, and may actually be null. In this latter scenario, replication comes with the same 5% risk of an alpha error as in the initial experiment.

## An unconventional “replication” experiment

**Fig 2:**
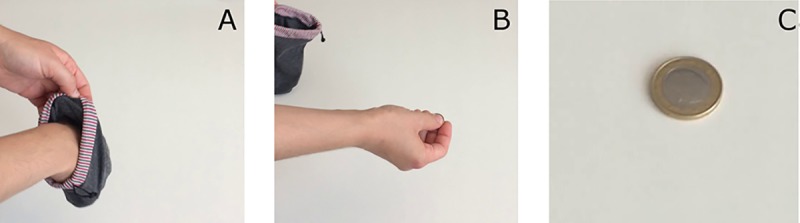
Results of the “replication” experiment. Screenshots of the coin flip experiment: (A) blind selection of coin and (B) flipping the coin (C) resulting in heads.

For our unconventional replication experiment, we used a fair coin and a single coin flip to attempt to replicate the effectiveness of VPA on lowering brain infarct volumes. Study plan and procedure of the replication experiment were preregistered [13], and further details are given in S1 Text. It was set a priori that if both observers judged the coin flip to have landed heads, the drug was deemed effective. The coin toss experiment took place on July 26, 2017, and was documented on video (https://www.youtube.com/watch?v=hhSbWARIEnM). Both observers agreed that the coin flip resulted in heads (Fig 2), indicating the replication of the protective VPA effect on brain infarct volume found in the initial study.

Clearly, we do not believe that a coin flip can help us to infer whether or not VPA has a beneficial neuroprotective effect. We use this absurd example to highlight the similarly absurd (from a frequentist probability perspective) exact replication scenario. In contrast to a coin toss, an exact replication would have consumed considerable resources with the suffering and death of 20 additional mice, but with no greater probability of replication than of our coin toss experiment (under the assumption that the initially observed effect equaled the population effect). Even if the initially observed *p* value had been substantially smaller than 0.05 (i.e., the estimate of the population effect had been considerably larger), the probability of successful replication at *p* < 0.05 would still have been low when using the same sample size. For example, the probability of replication with an initial *p* value of 0.01 would only have been 73% in this scenario. To achieve 95% probability of replicating a significant *p* value with an exact replication, the initial *p* value would have had to be *p* = 0.00032 [10]. These facts are well known to statisticians. Indeed, a coin flip example is often used in teaching the positive predictive value of a statistically significant result [14]. Notwithstanding, most scientists who are conducting, contemplating, or interpreting replication experiments are unaware of them and of their consequences. As absurd as our “replication” experiment appears, it has a serious kernel. We chose this approach because we hope to provoke readers to consider the idiosyncrasies of replication study designs and to perhaps spark discussion amongst colleagues. We discuss additional issues pertinent to replications in the current biomedical research context.

## Limitation of the binary approach of statistical testing in initial and replication experiments

A *p* value alone has limited value and provides little information about the underlying effect of interest [14–17]. This is true for any type of experiment, replication or otherwise.

Because treatment effects measured in small samples have larger variability around their corresponding population effects (relative to treatment effects estimated with larger sample sizes), their associated *p* values will likewise be less reliable. Geoff Cumming used simulations and animated graphics to illustrate the large stochastic variability of *p* values, simply because of sampling variability [18, 19]. If power of the initial study is low, then the *p* value of the second study with the same sample size is likely to vary substantially from that observed in the first study [15, 18]. Moreover, Miller and Schwarz showed that the statistical uncertainty of the initial observed effect often prevents accurate estimation of the replication probability and concluded that it is essentially impossible to predict whether a single statistically significant finding will replicate [20]. According to this view, replications should not only be evaluated by whether they are statistically significant or not—replications should not necessarily be characterized as successes or failures. Divergent statistical results in initial and replication experiments may occur, e.g., if statistical power is low in either one: a false positive might be observed by chance in the initial experiment, or a false negative in the replication [21]. There are various metrics for concluding that replications have or have not been successful and how to use them to interpret divergent results [22, 23].

## Need for an effect size estimate in both initial and replication experiments

Researchers are well advised to focus their research around the central question: “What is the effect (size)?” instead of the binary “Is there a statistically significant effect?” A *p* value is not solely a measure of an observed effect but depends on sample size as well; therefore, more information can be conveyed by reporting effect sizes [18]. Examining point estimates of effect sizes and measures of precision for both an initial experiment and its replication allows comparisons of the direction and of the strength of the observed effect in both studies. Effect size point estimates are less dependent on sample size and should be similar in both experiments if the replication was successful. Providing that the two experiments estimate the same underlying effect, providing point and precision estimates have the added advantage of allowing for Bayesian [24] or meta-analytic approaches [25], which combine evidence of the original (prior) with the new data (replication experiment) to gain more reliable evidence.

## Power considerations are pivotal for replication studies

Power considerations should be obligatory for both initial experiments and for their replications [15, 26, 27]. In order to reject the null hypothesis efficiently in a replication experiment, a (typically substantial) increase in sample size is necessary [28].

Simonsohn [29] looked at the replication problem the other way around and proposed to use 2.5× the sample size of the original experiment to obtain 80% power to reject the hypothesis of a detectable effect, e.g., an effect that the original sample had 33% power to detect, assuming the true effect is zero (note that the null hypothesis here is that there is an effect, and the alternative hypothesis is that there is no effect at all). Lakens as well as Neumann and colleagues suggested to use group sequential designs with stopping rules for success and futility, and calculation of *p* value and Bayes factor in parallel [30, 31]. Although sequential analyses aim to test hypotheses rather than to provide accurate effect size estimates, the latter can only be reached by larger sample sizes or meta-analyses [32].

To further explore and understand the role of power in replication experiments, we provide a web application in which initial sample size, initial results, and sample size of the replication experiment can be manipulated for determining power of a replication experiment under different scenarios (s-quest.bihealth.org/power_replication/). Fig 1 shows the power of the replication experiment depending on sample size and expected effect with our web application.

Three observations are noteworthy: (1) Assuming that the effect observed in our original experiment equals the population effect, an exact replication with the same sample size yields 52.5% power of detecting an effect with alpha set to 0.05, which motivated our coin flip analogy. (2) Under this same scenario, considerably larger sample sizes per group would have been necessary for a high-powered replication experiment (*n* = 25 for power = 91.1% and *n* = 50 for 99.7% power). (3) Under the assumption that the true population effect is zero, there is a 5% probability that the null hypothesis would nonetheless have been falsely rejected under all sample size scenarios, replicating the original experiment’s false positive finding (Type I error).

## Increased generalizability through conceptual rather than exact replication

Exact replications, regardless of using the same or an increased sample size compared with the initial experiment, can only show whether a certain effect can be replicated in a specific setting. However, to what extent the same effect can also be generalized can only be learned from replications that vary some aspect of the original design (e.g., different species, different laboratory, etc.) and thus increase the external validity of the results [2]. Such conceptual replications are particularly important when translating preclinical findings in animals to humans, because clinical trial in humans cannot exactly replicate animal studies.

In Table 1, we provide an overview of what additional information could be gained by performing an exact replication with or without an increase in power, as well as from conceptual replication, all compared to a coin toss. As argued above, an exact replication with the same sample size is potentially useful for identifying technical problems in the initial experiment, and the data can be used in meta-analytical or Bayesian approaches for more precise point estimates. By contrast, conceptual replication probes the robustness of results. If dissimilarities are observed between the initial experiment and the conceptual replication, however, it is unknown whether this is caused by false inference in the initial experiment (i.e., there is no true effect) or whether the two experiments are in fact addressing two different research questions, showing that there is value in performing both types of replications.

**Table 1:** Attributes and applications of different methods of replication.

Method of replication/ Attributes	Coin flip replication	Exact replication (same design, same sample size)	Exact replication with increased sample size (e.g., 2.5× sample size of initial study)	Conceptual replication (meaningful alterations to design, varying sample size)
Can identify technical mistakes in initial experiment	no	yes	yes	maybe
Can be used to reduce false inference on treatment effects	no	maybe	yes	maybe
Can provide information on robustness	no	no	no	yes
Can be used for meta-analyses	no	yes	yes	maybe

We used the apparently fallacious example of combining an animal experiment with a game of chance to illustrate and discuss the statistical challenges and complexities of replication experiments. We stress, however, that our argument here is not that exact replication does not have a role in the scientific process. In fact, it has a very useful but also limited and specific purpose. Although reproducibility is a complex construct [21, 33, 34], there appears to be consensus that replicability of results within one laboratory (“exact replication”) is an important element of the scientific method. All efforts should be made to make research replicable; i.e., the methodology should be thus designed and described that others (hypothetically) could repeat the experiment. An exact replication can increase the confidence in an experimental finding and rule out experimental, statistical, and other artifacts [2]. However, scientists, who with the best of intentions replicate their pivotal results with the same sample size, should be aware of the limitations of this procedure. Confidence in the results can be achieved in the first instance by exact replication (when possible) but with increased sample size to provide adequate power. Confidence in the robustness and generizability of results can be best achieved with adequately powered conceptual replications across multiple laboratories [35].

## Conclusions

We describe the design and results of a preregistered animal experiment to establish the efficacy of VPA to reduce brain infarct volumes in murine stroke, which we combine with a coin toss as a substitute for an exact replication. The absurd but true notion that a coin flip provides approximately the same positive predictive value as an exact replication experiment when the initial effect is barely significant highlights an important, but little known, limitation of exact replications. Although replication is a complex construct that eludes simple definition, we can learn from both successful and failed replication attempts [36], provided that we avoid underpowered initial and replication experiments. Moreover, effect sizes and corresponding measures of precision should be prioritized over reference to statistical significance.

## Supporting information

S1 Fig Results of the original experiment. Brain infarct volumes with and without treatment of VPA. *N* = 10 per group. Box plots represent median, 25th and 75th percentile, mean (dotted line), 5th and 95th percentile (whiskers) and, additionally, the individual data points that are also given in S1 Data.
(TIF)

S1 Data Data for Fig 1 and S1 Fig. (XLSX)

S1 Text Methods original and ‘replication’ experiment. (DOCX)

## Abbreviations

CI: confidence interval
MCAO: middle cerebral artery occlusion
ncp: noncentrality parameter
SD: standard deviation
VPA: valproic acid
