# Supplementary figures and images for: Exact replication: Foundation of science or game of chance?
Source: PLoS Biol. 2019 Apr 9;17(4):e3000188. doi: 10.1371/journal.pbio.3000188 (PMC6456162; doi:10.1371/journal.pbio.3000188)

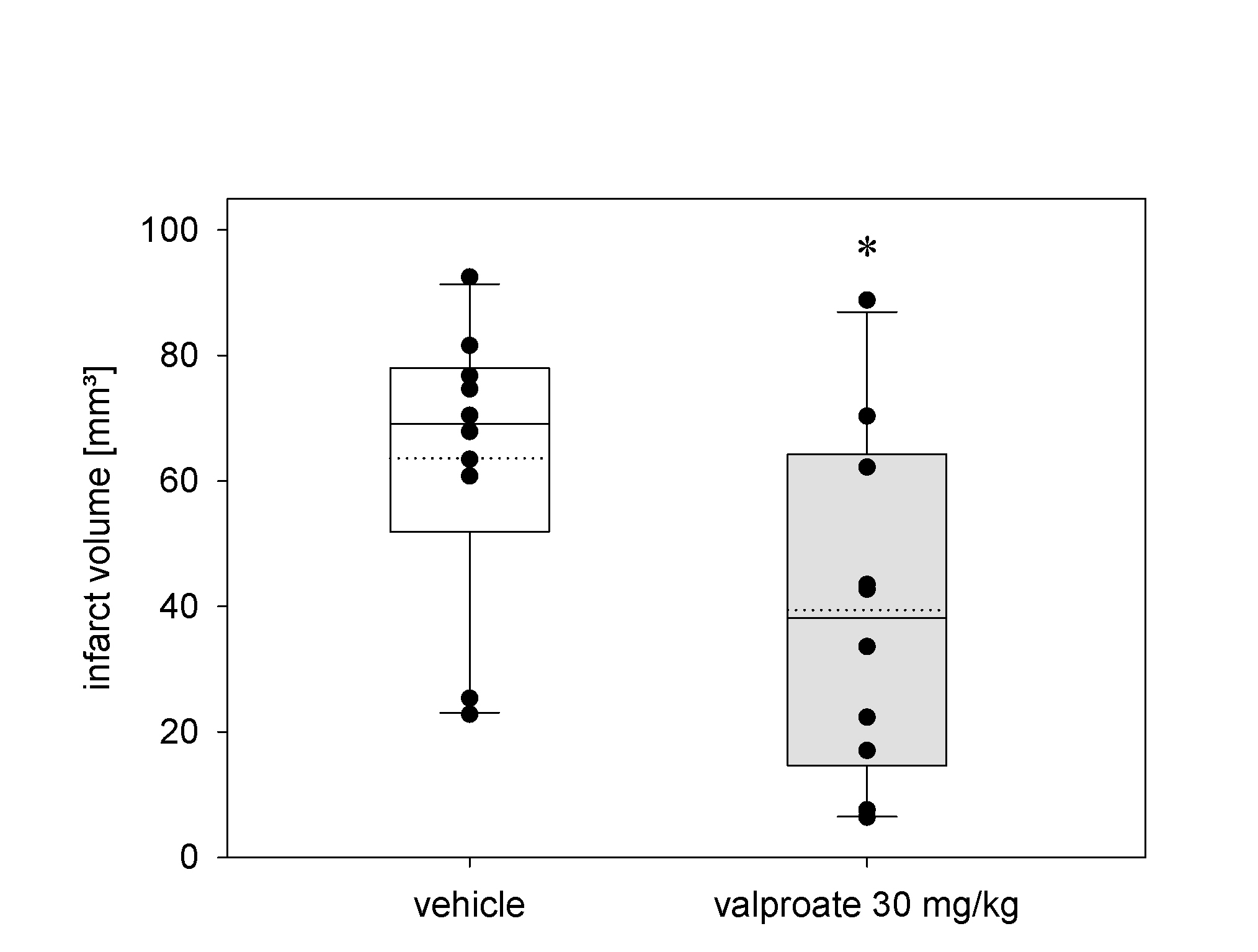

Supplement: S1 Fig — Brain infarct volumes with and without treatment of VPA. N = 10 per group. Box plots represent median, 25th and 75th percentile, mean (dotted line), 5th and 95th percentile (whiskers) and, additionally, the individual data points that are also given in S1 Data. (TIF) [file pbio.3000188.s001.tif]
