# Supplementary material for: Exact replication: Foundation of science or game of chance?
Source: PLoS Biol. 2019 Apr 9;17(4):e3000188. doi: 10.1371/journal.pbio.3000188 (PMC6456162; doi:10.1371/journal.pbio.3000188)
Supplement: S1 Text — (DOCX) [file pbio.3000188.s003.docx]

**Supporting Information**

**S1 Methods original experiment**

Twenty male C57Bl/6 N mice (drug naïve prior treatment, mean body weight: 24.3; SD: 0.8 g, Charles River Laboratories, Sulzfeld, Germany) were kept group housed (n=5 in a type 3 cage) under a 12 hours light-on/light-off regimen at 22±2°C with tap water and food (Ssniff, mouse standard diet) freely available. They underwent transient intraluminal MCAO for 45 minutes under inhalational anesthesia (1.5% isoflurane in 70% N_2_O and 30% O_2_) as previously described in our standard operating procedure for [middle cerebral artery occlusion in the mouse](http://precedings.nature.com/documents/3492/version/3/html) (Dirnagl and Group 2009).

VPA (30 mg/kg, i.p., Desitin, Hamburg, Germany) or vehicle was administrated in 10 animals each immediately after reperfusion, 12 hours later and then twice daily (every 12 hours) for 7 days, to reflect an optimal treatment regimen for neuroprotection.

Allocation of the animals to the treatment group or vehicle group was predetermined prior MCAO surgery using a computer-generated allocation table. Primary outcome of interest was brain infarct measured in mm^3^ and determined by support staff who were not aware of the treatment status. Infarct volume of both groups was compared with student’s t-test for independent groups, using a two-sided significance level of 0.05. Confidence intervals of the effect size were calculated using the package ‘*mbess’* in R Software (Kelley 2007).

All animal experiments, inclusive the welfare-related assessments and interventions that were carried out prior to, during, or after the experiment were performed according to protocols approved by the Berlin Authorities (ethics committee of the “Landesamt für Gesundheit und Soziales Berlin”, LaGeSo Reg 390/09).

**S1 Methods ‘replication’ experiment**

For this unconventional replication experiment, we used a fair coin and a single coin flip attempting to replicate the effectiveness of VPA on lowering brain infarct volumes. Study plan and procedure of the replication experiment were preregistered (Dirnagl, Grittner et al. 2017). A 1 euro coin was selected from a set of 5 sufficiently fair 1€ coins placed in an nontransparent bag by a person unaware of the purpose of the procedure, who then flipped the coin under the observation of two independent observers. The rule for the translation of the coin flip to a statement regarding treatment efficacy was set *a priori*: if both observers determined the coin flip landed heads, the drug was deemed effective. Conversely, agreement that coin flip landed tails resulted in the conclusion that treatment was not effective. The coin flip was planned to be performed only once unless the observers could not agree whether the coin landed heads or tails, or no outcome could be determined (e.g. coin slipped and was lost). Under one of these scenarios, the procedure would have been repeated with a fresh coin.
